# Dynamic response to peripheral nerve injury detected by in situ hybridization of IL-6 and its receptor mRNAs in the dorsal root ganglia is not strictly correlated with signs of neuropathic pain

**DOI:** 10.1186/1744-8069-9-42

**Published:** 2013-08-16

**Authors:** Václav Brázda, Ilona Klusáková, Ivana Hradilová Svíženská, Petr Dubový

**Affiliations:** 1 Central European Institute of Technology (CEITEC), Masaryk University, Kamenice 3, 62500, Brno, Czech Republic; 2 Institute of Biophysics, Academy of Sciences of the Czech Republic v.v.i., Královopolská 135, 61265, Brno, Czech Republic; 3 Department of Anatomy, Division of Neuroanatomy, Faculty of Medicine, Masaryk University, Brno, Czech Republic

**Keywords:** Cytokines, Contralateral reaction, IL-6 signaling, Neuroinflammation, Neuropathic pain, Unilateral nerve injury

## Abstract

**Background:**

IL-6 is a typical injury-induced mediator. Together with its receptors, IL-6 contributes to both induction and maintenance of neuropathic pain deriving from changes in activity of primary sensory neurons in dorsal root ganglia (DRG). We used in situ hybridization to provide evidence of IL-6 and IL-6 receptors (IL-6R and gp130) synthesis in DRG along the neuraxis after unilateral chronic constriction injury (CCI) of the sciatic nerve as an experimental model of neuropathic pain.

**Results:**

All rats operated upon to create unilateral CCI displayed mechanical allodynia and thermal hyperalgesia in ipsilateral hind paws. Contralateral hind paws and forepaws of both sides exhibited only temporal and nonsignificant changes of sensitivity. Very low levels of IL-6 and IL-6R mRNAs were detected in naïve DRG. IL-6 mRNA was bilaterally increased not only in DRG neurons but also in satellite glial cells (SGC) activated by unilateral CCI. In addition to IL-6 mRNA, substantial increase of IL-6R mRNA expression occurred in DRG neurons and SGC following CCI, while the level of gp130 mRNA remained similar to that of DRG from naïve rats.

**Conclusions:**

Here we evidence for the first time increased synthesis of IL-6 and IL-6R in remote cervical DRG nonassociated with the nerve injury. Our results suggest that unilateral CCI of the sciatic nerve induced not only bilateral elevation of IL-6 and IL-6R mRNAs in L4–L5 DRG but also their propagation along the neuraxis to remote cervical DRG as a general neuroinflammatory reaction of the nervous system to local nerve injury without correlation with signs of neuropathic pain. Possible functional involvement of IL-6 signaling is discussed.

## Background

Cytokines and their receptors are involved in many biological processes, including rapid responses of the nervous system to injury inducing and maintaining neuropathic pain. An injury to peripheral nerve initiates a cascade of cellular and molecular changes involving neurons, glial cells, and immune cells [1,2]. Neuronal cell functions in the dorsal root ganglia (DRG) shift from normal maintenance and neurotransmission toward survival and regeneration after peripheral nerve injury. The DRG changes after nerve injury are related to up- and down-regulation of proteins, rapid modulation of satellite glial cells (SGC) activity, invasion of macrophages [3,4], and cytokine expression [5,6].

Interleukin 6 (IL-6) serves as a pleiotropic cytokine in various tissue systems, depending on the target cell and actual cell conditions [7]. In the nervous system, IL-6 contributes to the survival of axotomized neurons, growth and differentiation of neurons, formation of dendrites, neurotransmitter metabolism, and other processes [8,9]. IL-6 is able to promote axonal regeneration [10], but there is evidence that it contributes also to the development of neuropathic pain [5,11]. IL-6 mRNA is scarcely detected in intact sciatic nerve [12], DRG [13], and the spinal cord [5], but it is increased following nerve injury [13]. It has been shown that some cytokines, chemokines and their receptors are induced bilaterally in lumbar DRG [14,15] and also in remote cervical DRG after unilateral sciatic nerve injury [15,16].

IL-6 signals are mediated through a cell-surface cytokine receptor complex consisting of the ligand-binding IL-6Rα chain and the signal-transducing component gp130. gp130 functions as the common receptor for a functionally and structurally similar family of cytokines including IL-6, leukemia inhibitory factor (LIF), ciliary neurotrophic factor (CNTF), oncostatin M, cardiotrophin-1, and IL-11 [17]. All sensory neurons express gp130 immunoreactivity, and its levels and intracellular distribution remain unchanged up to 14 d following sciatic nerve axotomy [14,18]. Real-time polymerase chain reaction has revealed a higher expression of gp130 within the superior cervical ganglia after axotomy for three members of this cytokine family (IL-6, IL-11, and LIF) that is accompanied by modest increases in oncostatin M, no changes in CNTF, and decreases in cardiotrophin-1 [19]. Naïve lumbar DRG have shown immunofluorescence staining for IL-6R in small and medium-sized neuronal bodies while SGC displayed no distinct IL-6R immunoreaction. Unilateral CCI of the sciatic nerve for 3, 7 and 14 d has been shown to induce an enhanced IL-6R immunofluorescence in SGC surrounding neuronal somata of L4–L5 DRG ipsilateral to nerve injury [20].

Sciatic nerve ligature is frequently used as a model for nerve degeneration/regeneration as well as neuropathic pain. The primary sensory neurons are affected directly by nerve injury or indirectly by activated SGC and adjoining immune cells that release a variety of molecules changing the microenvironment of the neurons [21,22]. The majority of neuropathic pain models currently in use share alternations in hind-limb cutaneous sensory thresholds following partial injury of the peripheral nerve as a common feature. The CCI model is well described and is associated with the development of spontaneous pain-like behavior [23].

The aim of our study was to determine mRNA synthesis of IL-6 and its receptors IL-6R and gp130 in DRG associated and nonassociated with peripheral nerve injury in order to explain its existence and spreading after CCI of the sciatic nerve as an experimental model of neuropathic pain. We show that the inflammatory response to neuropathic stress is propagated quickly on the expression level from the lumbar to outlying cervical DRG, but without significant changes of mechanical allodynia and thermal hyperalgesia in forepaws.

## Results

### Behavioral tests

**Figure 1:**
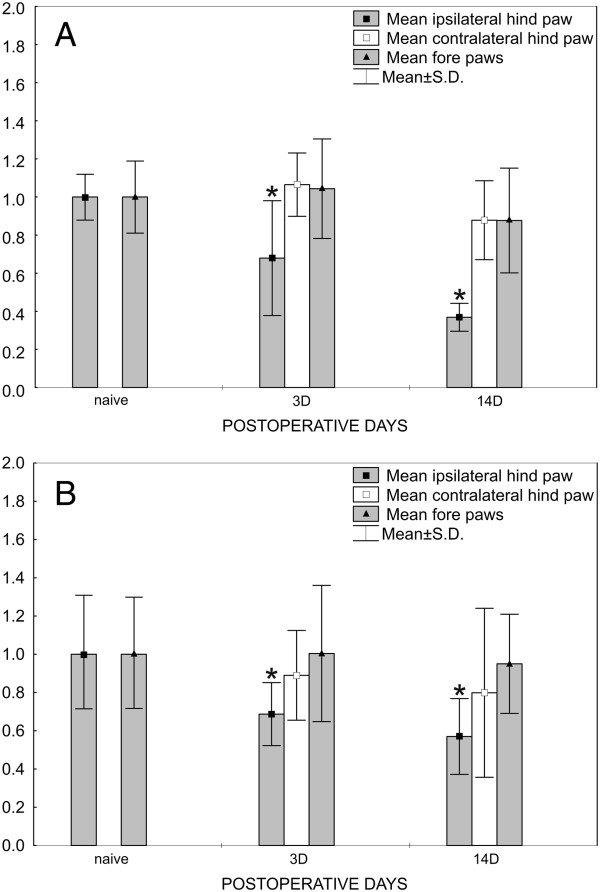
**Results from behavioral tests.** Data are for rats 1 d before operation and the same rats operated upon to create unilateral CCI of the sciatic nerve for 3 and 14 d. Progressive development of evoked mechanical allodynia **(A)** and thermal hyperalgesia **(B)** was found in the ipsilateral hind paws. No significant changes of mechanical allodynia and thermal hyperalgesia were observed in either contralateral hind paws or forepaws. Data are expressed as relative change of values in comparison to values measured 1 d before operation, such that a value of 1 indicates no change. *Indicates statistically significant difference (p < 0.05) when compared with measurement 1 d before operation.

All rats operated upon to create CCI of the sciatic nerve and used for in situ hybridization (ISH) displayed decreased withdrawal threshold for mechanical allodynia (Figure 1A) and thermal hyperalgesia (Figure 1B) in hind paws ipsilateral to nerve ligatures, thus indicating neuropathic pain at 3 d and 14 d after operation. Contralateral hind paws and forepaws of both sides did not exhibit statistically significant changes of withdrawal thresholds for mechanical allodynia and thermal hyperalgesia when compared with 1 d before operation. Sham-operated rats did not exhibit mechanical allodynia or thermal hyperalgesia either ipsilaterally or contralaterally to CCI (not shown).

### IL-6 mRNA in DRG of naïve, CCI- and sham-operated rats

**Figure 2:**
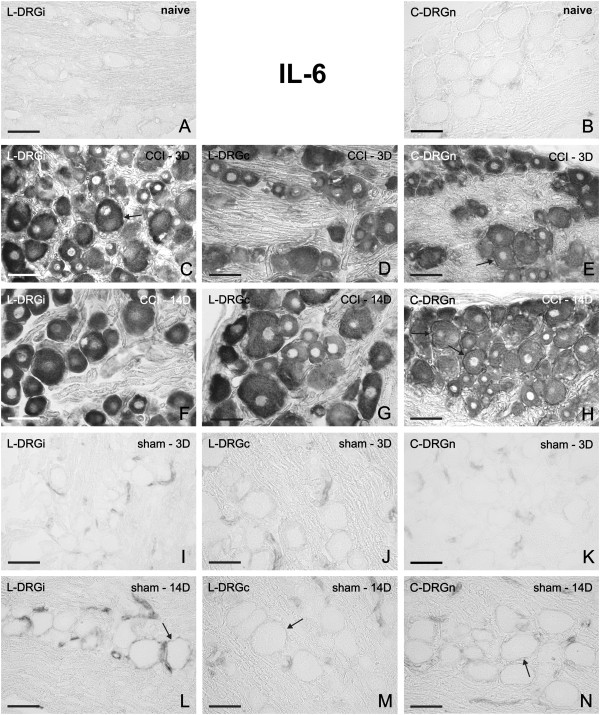
**Representative ISH staining of IL-6 mRNA signal.** Detection of IL-6 mRNA in sections of lumbar ipsilateral DRG (L-DRGi, first column, **A**, **C**, **F**, **I**, **L**), lumbar contralateral DRG (L-DRGc, second column, **D**, **G**, **J**, **M**), and cervical DRG (C-DRGn, third column, **B**, **E**, **H**, **K**, **N**). Naïve DRG (first row, **A**, **B**), and DRG from CCI- (second and third rows, **C**–**H**) and sham-operated (fourth and fifth rows, **I**–**N**) rats. Arrows indicate position of SGC. Scale bars = 50 μm.

No or very weak signals for IL-6 mRNA were observed in sections of DRG removed from the naïve rats. A weak staining for IL-6 mRNA was detected in SGC and the blood vessels of both lumbar and cervical DRG (Figure 2A, B). A bilateral elevation of staining for IL-6 mRNA was induced in lumbar DRG by unilateral CCI of the sciatic nerve for 3 d (Figure 2C,D). Surprisingly, we observed also bilateral elevation of staining for IL-6 mRNA in cervical DRG (Figure 2E). A similar pattern of IL-6 mRNA staining was also found bilaterally in both lumbar and cervical DRG 14 d after CCI (Figure 2F–H). In comparison to naïve DRG, a distinct intensity of IL6 mRNA staining was observed, particularly in neuronal somata of all sizes and in their SGC. Quantitative densitometry analysis of DRG neurons with SGC for IL-6 mRNA staining is summarized in Table 1 (third column). There were significant differences (p < 0.05) in the staining for CCI-operated animals in both lumbar and cervical DRG when compared with naïve DRG. Intense staining for IL-6 mRNA in neuronal bodies did not usually allow to distinguish staining in the SGC, which was possible particularly in SGC surrounding the large neuronal bodies.

**Table 1:** Staining density for IL-6, IL6R, and gp130 mRNA

**Mean density (D ± SD)**				
Animals	Ganglia and time	IL-6	IL-6R	Gp130
Naïve	L-DRGn	0.053 ± 0.006	0.170 ± 0.050	0.320 ± 0.121
	C-DRGn	0.042 ± 0.006	0.142 ± 0.042	0.440 ± 0.755
Operated	3D L-DRGi	0.386 ± 0.139*	0.434 ± 0.111*	0.540 ± 0.090
	3D L-DRGc	0.427 ± 0.064*	0.405 ± 0.119*	0.450 ± 0.047
	3D C-DRGn	0.464 ± 0.069*	0.518 ± 0.119*	0.490 ± 0.394
	14D L-DRGi	0.536 ± 0.070*	0.273 ± 0.065*	0.470 ± 0.192
	14D L-DRGc	0.419 ± 0.132*	0.165 ± 0.030	0.250 ± 0.182
	14D C-DRGn	0.332 ± 0.079*	0.201 ± 0.046	0.440 ± 0.154
Sham-operated	3D L-DRGi	0.045 ± 0.006	0.241 ± 0.053	0.370 ± 0.276
	3D L-DRGc	0.062 ± 0.006	0.232 ± 0.030	0.270 ± 0.089
	3D C-DRGn	0.056 ± 0.006	0.207 ± 0.062	0.320 ± 0.459
	14D L-DRGi	0.075 ± 0.010	0.208 ± 0.033	0.290 ± 0.544
	14D L-DRGc	0.074 ± 0.007	0.159 ± 0.015	0.340 ± 0.058
	14D C-DRGn	0.085 ± 0.005	0.210 ± 0.040	0.490 ± 0.309

*Statistical significance (p < 0.05) when compared with naïve DRG.

Results of staining density for mRNA in lumbar ipsilateral (L-DRGi), lumbar contralateral (L-DRGc), and cervical (C-DRGn) dorsal root ganglia removed from naïve (Naïve), CCI- and sham-operated rats for 3 and 14 d of survival (3D, 14D).

By contrast, sections of DRG removed from rats 3 d (Figure 2I–K) or 14 d (Figure 2L-N) after sham-operation displayed moderate IL-6 mRNA staining in SGC that predominantly surrounded large neurons. A higher intensity of IL-6 mRNA staining was observed in SGC of DRG removed at 14 d (Figure 2L–N) than at 3 d (Figure 2I–K) from sham operation. However, quantitative analysis of neurons with SGC for IL6 mRNA staining did not show significant changes in the staining within DRG from sham-operated animals when compared to naïve DRG (Table 1). Control sections incubated without any probe and with sense oligonucleotide probes displayed no color staining (Additional file 1A–C).

In parallel with ISH, we analyzed changes of IL-6 protein in DRG. No or very weak immunofluorescence for IL-6 protein was observed in sections of DRG removed from the naïve rats (Additional file 2A). Increased staining for IL-6 protein was induced in both lumbar and cervical DRG by unilateral CCI of the sciatic nerve for 3 d (Additional file 2B–D) and for 14 d (not shown). The results of IL-6 protein elevation by in situ proteomics are in agreement with the increased corresponding mRNA signals detected by ISH.

### IL-6R and gp130 mRNAs in DRG of naïve, CCI- and sham-operated rats

**Figure 3:**
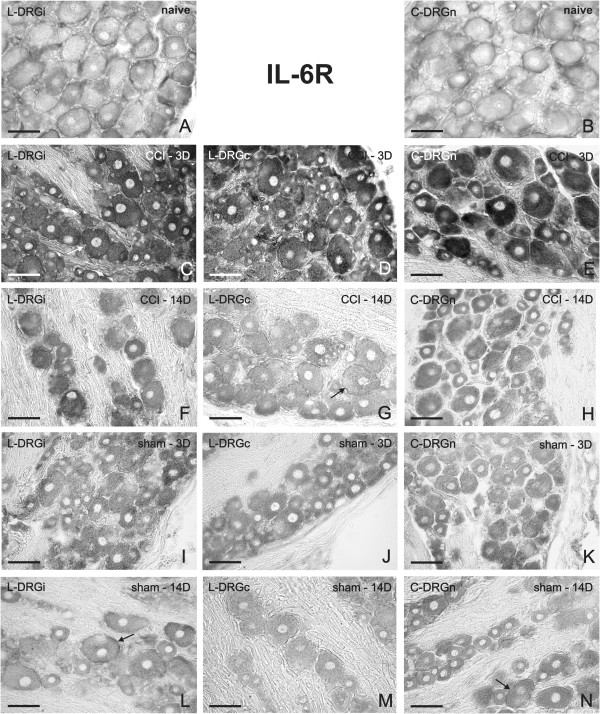
**Representative ISH staining of IL-6R mRNA signal.** Detection of IL-6R mRNA in sections of lumbar ipsilateral DRG (L-DRGi, first column, **A**, **C**, **F**, **I**, **L**), lumbar contralateral DRG (L-DRGc, second column, **D**, **G**, **J**, **M**), and cervical DRG (C-DRGn, third column, **B**, **E**, **H**, **K**, **N**). Naïve DRG (first row, **A**, **B**), and DRG from CCI- (second and third rows, **C**–**H**) and sham-operated (fourth and fifth rows, **I**–**N**) rats. Arrows indicate position of SGC. Scale bars = 50 μm.

A weak signal for IL-6R mRNA was observed in neuronal bodies and SGC in sections of both lumbar and cervical DRG removed from the naïve rats (Figure 3A, B). Bilateral elevation of staining for IL-6R mRNA was induced in neuronal bodies of all sizes and SGC of lumbar (Figure 3C,D) and cervical (Figure 3E) DRG after unilateral CCI of the sciatic nerve, and especially after 3 d. The elevation of IL-6R mRNA was not so strong at 14 d from CCI (Figure 3F–H). The pattern and intensity of staining for IL-6R mRNA were very similar in both cervical and lumbar DRG sections for each period of survival. Quantitative analysis of neurons with SGC for density of IL-6R mRNA staining is summarized in Table 1 (fourth column). There were significant differences (p < 0.05) in the staining for CCI-operated animals only 3 d after operation in both lumbar and cervical DRG when compared with naïve DRG. Even though we observed some changes 14 d after operation, significant differences were measured only in ipsilateral lumbar DRG at this time. However, sections of DRG removed at 3 d (Figure 3I–K) or 14 d (Figure 3L–N) from sham-operated rats displayed IL-6R mRNA staining comparable to the signal in naïve rats (Figure 3A,B). Modest increase of IL-6R mRNA staining without statistical significance was detected in neurons and SGC by quantitative analysis for sections of DRG removed at 3 d or 14 d from sham-operated rats (Table 1). We analyzed also changes of IL-6R protein in DRG in relation to IHC. While in DRG of naïve rats a very weak signal for IL-6R protein was detected (Additional file 3A), a considerable elevation of immunofluorescence for IL-6R protein was observed in both lumbar and cervical DRG from rats operated on CCI for 3 d (Additional file 3B–D). These results from protein analyses are in agreement with our ISH results.

**Figure 4:**
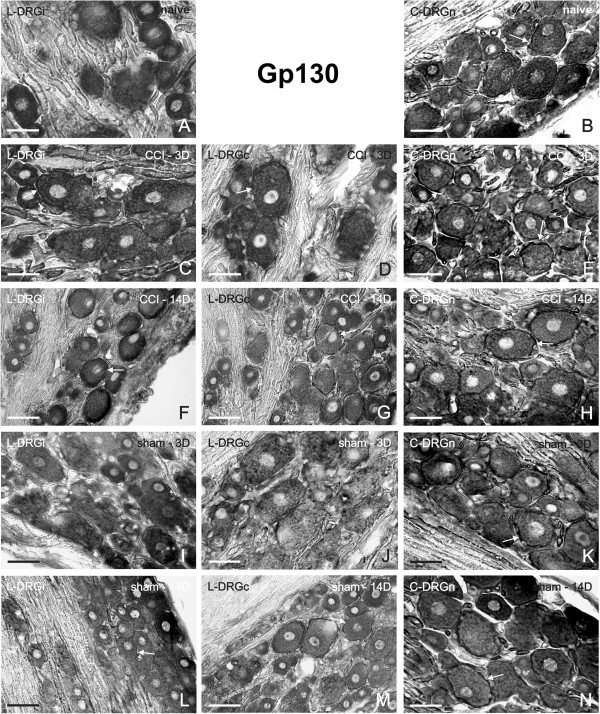
**Representative ISH staining of gp130 mRNA signal.** Detection of gp130 mRNA in sections of lumbar ipsilateral DRG (L-DRGi, first column, **A**, **C**, **F**, **I**, **L**), lumbar contralateral DRG (L-DRGc, second column, **D**, **G**, **J**, **M**), and cervical DRG (C-DRGn, third column, **B**, **E**, **H**, **K**, **N**). Naïve DRG (first row, **A**, **B**), and DRG from CCI- (second and third rows, **C**–**H**) and sham-operated (fourth and fifth rows, **I**–**N**) rats. Arrows indicate position of SGC. Scale bars = 50 μm.

In contrast to IL-6 and IL-6R mRNA, we observed intense signal for gp130 mRNA in sections of DRG removed from the naïve rats, and especially in neuronal bodies and SGC (Figure 4A,B). A similar pattern of staining was observed also in operated (Figure 4C–H) and sham-operated (Figure 4I–N) animals in all analyzed lumbar and cervical DRG. Densitometry of neurons with SGC for gp130 mRNA staining is summarized in Table 1 (fifth column). Although quantitative evaluation showed slight changes in the intensity of signals, there were no significant differences between the signals at any of the tested times. The control sections incubated with sense oligonucleotide probes for both IL-6R (Additional file 1D–F) and gp130 (Additional file 3G–I) displayed no color staining.

### Double-staining by ISH and GFAP immunostaining

**Figure 5:**
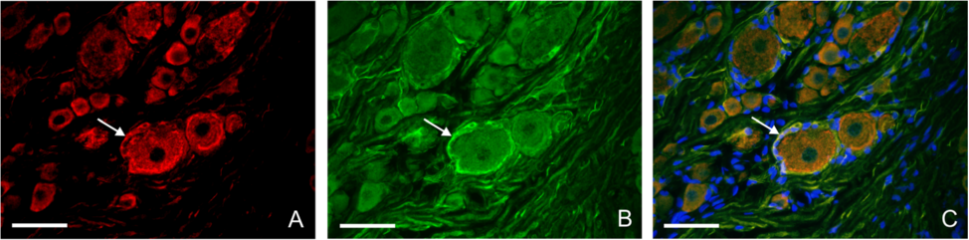
**Representative double-staining of IL-6 mRNA by ISH and immunofluorescence for glial fibrillary acidic protein (GFAP).** Detection of IL-6 mRNA in section of ipsilateral L4 DRG from sham-operated rat 14 d after surgery **(A)** and simultaneous immunofluorescence staining for GFAP **(B)**. Signal for IL-6 mRNA was co-localized with GFAP immunofluorescence in SGC **(C)**. The nuclei were labeled by Hoechst 33342. Arrows indicate position of SGC. Scale bars = 50 μm.

The presence of IL-6 mRNA in SGC was verified by double-staining for IL-6 mRNA. Glial fibrillary acidic protein (GFAP) was used as a marker of SGC. The co-localization of IL-6 mRNA with GFAP immunostaining was evident particularly at SGC surrounding large neuronal bodies (Figure 5A–C).

## Discussion

Cytokines play a crucial role as intercellular messengers of immune reaction to nervous system injury [24,25]. Under normal conditions, the production of cytokines aids the immune system in reaction to pathogens and healing damaged tissue. On the other hand, prolonged pro-inflammatory cytokine release can induce or facilitate neuropathic pain [26]. Neuropathic pain refers to a variety of chronic pain conditions with differing underlying pathophysiological mechanisms and origins. Glial cells contribute importantly to the creation of enhanced pain states [27]. Following peripheral nociceptive activation via nerve injury, microglia become activated, release pro-inflammatory cytokines, and thereby initiate the pain process. Microglia propagates neuroinflammation by recruiting other microglia and eventually activating nearby astrocytes, which prolongs the inflammatory state and leads to a chronic neuropathic pain condition. [28]. Current therapies for pain management include the pervasive utilization of opioids [29]. Activated glial cells disrupt the pain-suppressive effects of opioid drugs and contribute to opioid tolerance and opioid dependence/withdrawal [30].

IL-6 is a pleiotropic cytokine with a diverse range of actions, including modulation of the peripheral and central nervous systems as well as several post-injury effects. CCI of the sciatic nerve induces neuropathic pain and synthesis of IL-6 in DRG [13,20]. It also has been demonstrated that administration of IL-6 neutralizing antibody significantly decreases allodynia [31]. Targeting of IL-6 signaling pathways in injured nerve may provide a novel therapeutic window for treating neuropathic pain associated with nerve injury [32].

Interleukin-6 mRNA has been found in the primary sensory neurons of rat DRG after nerve transection or constriction [13,33]. Available data show that IL-6 transcription is induced specifically in DRG cells, even if retrograde transport from the periphery cannot be excluded [34,35]. Our results revealed that IL-6 mRNA was increased not only in DRG affected by nerve injury but also in the contralateral DRG. Moreover, we observed bilateral increased staining for IL-6 mRNA also in cervical DRG nonassociated with nerve subjected to CCI. The presence of IL-6 protein and gp130 protein in lumbar DRG had previously been confirmed also by western blot analyses [14,36]. Our results are in accordance with a growing body of evidence that unilateral nerve injury evokes bilateral responses [15,16,37], as well as with bilateral propagation of neuroinflammatory reaction in DRG along the neuraxis from the spinal segments related to segments nonrelated to injured nerve [16,38]. In addition to its occurrence in the neurons, immunohistochemical staining has revealed IL-6 protein also in SGC [20]. Our results from double-staining of ISH for IL-6 mRNA and GFAP immunostaining confirmed the synthesis of IL-6 not only in the DRG neurons but also in their SGC. Interleukin 6 signals through a cell-surface cytokine receptor complex consisting of the ligand-binding IL-6Ra chain and the signal-transducing component gp130. Alternatively, IL-6 may act via a soluble form of the IL-6R generated by limited proteolysis of the membrane-bound receptor or by alternative splicing of IL-6R mRNA [39]. Some experimental results have suggested that IL-6R is not synthesized by DRG neurons [40]. Our experiments reported here revealed significant elevation of IL-6R mRNA staining in neuronal bodies of all sizes as well as in SGC surrounding mainly large neurons of DRG removed from CCI-operated rats when compared with those of naïve rats. This is in agreement with our previously published results of immunostaining for IL-6R protein in DRG after CCI of the sciatic nerve [20]. Expression of gp130 mRNA and protein has been described in neuronal bodies rather than SGC of the rat DRG [18]. In accordance with our previous results [14], however, we detected gp130 mRNA expression in both neuronal bodies and SGC of DRG and its unaltered levels after CCI. This indicates that the basic level of gp130 is sufficient for signaling in response to IL-6 elevation after neuropathic stress. Moreover, gp130 is also an important receptor for other ligands, such as LIF, CNTF, oncostatin M, cardiotrophin-1, and IL-11. Similar changes of IL-6 and IL-6R mRNAs found bilaterally in both lumbar and cervical DRG following unilateral CCI of the sciatic nerve suggest their common principal regulation.

What signaling is involved in extending DRG reaction to heteronymous levels has not yet been clearly elucidated. The neuronal signaling of physiological imbalance induced by unilateral nerve injury could be transferred to homonymous contralateral or heteronymous DRG of both sides by neuronal activity along the neuronal pathways (e.g., through interneurons at the spinal cord segment or supraspinal levels) [41]. It is also known that ascending propriospinal systems link lumbar and cervical spinal cord segments [42]. This mechanism of propagating mRNA changes in DRG from lumbar to cervical segments is supported by findings that the expression of IL-6 mRNA in neurons is regulated by membrane depolarization [43]. Thus, an increased level of IL-6 mRNA in heteronymous DRG nonassociated with damaged nerve might be explained by transmission of neuronal activity from lumbar to cervical segments. Moreover, our findings of bilateral changes in TNF-α, IL-10, and CB-2 protein levels in DRG homonymous and heteronymous with damaged nerve indicate that transfer via the bloodstream cannot be excluded [16,44]. The blood flow is probably another route for dissemination of the signaling molecules from injured nerve stump to the proximity of DRG neurons not only associated but also nonassociated with the damaged nerve [15,16,38]. Peri-sciatic injection of selective inhibitors/antagonists has revealed that a number of immune products are early mediators of the resultant allodynias, including proinflammatory cytokines, reactive oxygen species, and complement. Thus, these immune-derived substances can markedly alter sensory nerve functions [45]. Many clinical pain syndromes are associated with mirror-image allodynia [46]. Although our experiments did not confirm allodynia in the contralateral segments, it can be assumed that some cytokines participate in initiating these processes.

In our experiments, we applied the sciatic nerve ligation using a 3–0 sterilized thread (Ethicon) under aseptic conditions to study neuropathic pain induction and levels of IL-6, IL-6R, and gp130 mRNAs in DRG. Therefore, the changes of mRNA levels in the ipsi- and contralateral L4–L5 as well as C7–C8 DRG were largely induced by traumatic nerve injury as a manifestation of accompanying neuroinflammatory response. Notable differences in IL-6 and IL-6R mRNAs were found between DRG from operated and sham-operated animals. With low staining for IL-6 and IL-6R mRNAs in neuronal bodies, a mild but distinct expression was observed in SGC of DRG from sham-operated animals. This activation of SGC in DRG after sham operation is supported by findings that skin incision led to proliferation of SGC [47] and increased expression of *ATF3* and other regeneration-associated genes in DRG [48]. We assume that even under aseptic conditions, sham operation without nerve injury leads to local production of tissue injury signals that spread via the bloodstream to DRG where they may activate SGC. This is supported by evidence that DRG are free of blood–nerve barrier [49].

While it is broadly accepted that pro-inflammatory cytokines are involved in induction of neuropathic pain [50,51], the particular role of IL-6 is a subject of controversy. It has been shown that exogenous administration of IL-6 is sufficient to stimulate nociceptors and cause pain [11,52,53]. It has been reported, however, that an increase in plasma IL-6 did not induce a hyperalgesic effect that would indicate IL-6 effects in the nervous structures, e.g., in DRG or the spinal cord [53,54]. In contrast to exogenous administration, endogenous IL-6 of the spinal cord can inhibit nociceptive transmission in neuropathic rats and thereby be a potential modulator of neuropathic pain [55,56]. Our results showing IL-6 and IL-6R up-regulation in lumbar DRG ipsilateral to nerve injury do not exclude the possibility that IL-6 signaling plays a role in inducing the behavioral signs of neuropathic pain. On the other hand, it should be stressed that additional molecular mechanisms are needed to induce neuropathic pain.

However, increased IL-6 and IL-6R mRNA levels were found also in contralateral lumbar and cervical DRG while not correlating with signs of neuropathic pain. This significant expression of IL-6 and IL-6R mRNAs in DRG nonassociated with injured nerve suggests other functional involvement of IL-6 signaling, such as a general neuroinflammatory reaction of the nervous system to injury. For example, IL-6 plays a role in promoting neuronal survival and axonal growth by DRG neurons [40,57,58]. In addition, it has been demonstrated that primary lesion promotes axon regeneration in contralateral nerve [37,59]. Therefore, we suppose that increased IL-6 and IL-6R mRNA in the primary sensory neurons of DRG nonassociated with damaged nerve might also be related to their conditioning through reactivation of an intrinsic growth program to regenerate their axons.

## Conclusions

No detectable or very low signals for IL-6 and IL-6R mRNAs were observed in neuronal bodies and their SGC of DRG from naïve rats. Unilateral CCI of the sciatic nerve induced a bilateral increase of staining for IL-6 and IL-6R mRNAs in neuronal bodies and SGC of both lumbar and cervical DRG while mechanical allodynia and thermal hyperalgesia were regularly measured only in hind paws ipsilateral to the injured nerve. In contrast, the gp130 mRNA level did not change in the neuronal bodies and SGC in DRG from naïve versus CCI-operated rats.

The data provide evidence for increase of IL-6 and IL-6R mRNA levels not only in DRG directly associated with damaged nerve but also in those nonassociated with the injured nerve of the experimental neuropathic pain model. The results suggest effective propagation of signal molecules along the neuraxis to remote cervical DRG as a general neuroinflammatory reaction of the nervous system to local nerve injury.

## Methods

### Animals and surgical procedures

Thirty pathogen-free Wistar rats (male, 250–300 g) used for the experiments were housed on a 12 h light/dark cycle at temperature 22–24°C under specific pathogen-free conditions in the animal housing area of Masaryk University. Sterilized standard rodent food and water were available ad libitum. All experimental procedures were carried out aseptically and according to protocols approved by the Animal Investigation Committee of the Faculty of Medicine, Brno, Czech Republic and followed ethical guidance [60].

Surgical procedures were performed under deep anesthesia using equal volumes of ketamine (40 mg/ml) and xylazine (4 mg/ml) solutions administered intraperitoneally (0.2 ml/100 g body weight). To create CCI, the right sciatic nerve was exposed at mid-thigh level by blunt dissection just proximal to its trifurcation, and three ligatures (3–0 Ethicon) were tied around the nerve with 1 mm spacing to reduce nerve diameter by approximately one-third. Animals operated upon to create CCI were left to survive for 3 or 14 d, representing the early and later period of survival. DRG removed from intact rats were used as naïve controls. The right sciatic nerves of sham-operated rats were only exposed without any nerve lesion and animals were also allowed to survive for 3 or 14 d.

### Behavioral tests

To ensure rats developed clear signs of neuropathic pain, mechanical allodynia and thermal hyperalgesia were measured in hind paws and forepaws before and after operation.

Mechanical allodynia was measured on a Dynamic Plantar Aesthesiometer apparatus (Ugo Basile, Comeria, Italy) according to the manufacturer’s manual. For testing, each rat was placed into a Plexiglas box with metal grid bottom. After 15 min of acclimatization there was applied to its paws rigid Von Frey filament with diameter 0.5 mm to a pressure of 50 grams. Threshold responses of paws were measured 4 times for each paw with 5 min intervals between tests 1 d before operation and at the final day of each survival period.

Thermal hyperalgesia was measured on a Plantar Test Instrument (Ugo Basile) according to the manufacturer’s manual. Each rat was placed in a Plexiglas box with plastic bottom. After 15 min of acclimatization a heat beam was applied to its paws. The intensity of radiance was set at value 50. The value of withdrawal latency was measured 4 times for each paw with 5 min intervals between tests 1 d before operation and at the end of each survival period. Maximum measurement time was 30 s to prevent tissue damage.

Mean values of behavioral measurement obtained for all animals 1 d prior to surgery were set as 1. Data for mechanical allodynia and thermal hyperalgesia after operation were expressed as relative changes in sensitivity of the same animal from 1 d before operation. Behavioral data were evaluated using Kruskal–Wallis one-way analysis of variance and p values less than 0.05 were considered to be significant. There were no statistically significant differences in sensitivity between left and right forepaws, so we analyzed results of forepaws sensitivity as a single set.

### In situ hybridization (ISH)

For DRG tissue harvesting, four naïve rats, the next four rats operated upon to create CCI for each period of survival (3 and 14 d), and sham-operated rats for 3 (n = 4) and 14 (n = 4) days were deeply anesthetized with a lethal dose of sodium pentobarbital (70 mg/kg body weight, i.p.) and perfused transcardially with 500 ml phosphate buffered saline (PBS, 10 mM sodium phosphate buffer, pH 7.4, containing 0.15 M NaCl and 0.1% diethylpyrocarbonate [DEPC]) followed by 500 ml of 4% paraformaldehyde containing 0.1% DEPC. The DRG samples were then washed in 20% phosphate buffered sucrose for 12 h and blocked in Tissue-Tek® OCT compound (Miles, Elkhart, IN, USA). Serial longitudinal cryostat sections (12 μm) through the DRG were mounted on chrome-alum covered slides.

To localize gene transcripts, ISH was performed according to the protocol of Harnicarova et al. [61]. We used 50-mer oligoprobes synthesized for the target gene transcripts. Digoxigenin (DIG)-dT was used for probe labeling, the sites of which are indicated by asterisks. Sequences of oligoprobes were as follow: IL-6 - (1) 5′DIG- CGC TGT TCA T AC AAT* CAG AAT TGC CAT* TGC ACA ACT CT*T T TCT CAT TTC C-3′DIG; (2) 5′DIG- TCA AGT GCT TTC AAG AT*G AGT TGG ATG GTC TTG GT*C CTT AGC CAC TCC TTC -3′DIG; Il-6R - 5′ DIG-AGT GTG TTT CCC GT*G GTA GTC CAT TCT CTG CT*C TGT GAG CCT GAG TAC 3′; and gp130 - (1) 5′ DIG-CGC TTT GGA TGG T*CT GTC TTC AT*A TGT GGT CCC GCT* CGC CTC CTC ACT 3′; (2) 5′ DIG-TCC CAG CAG CGT* TGT CAG GAG GAA AGC T*A AGC ACA CAG GCA CGA CTA T 3′ (VBC-Biotech, Vienna, Austria). All solutions used in this procedure were prepared in double-distilled water treated with DEPC. Digoxigenin was detected by DIG Colorimetric Nucleic Acid Detection Kit (Roche). The sections were mounted in a Vectashield aqueous mounting medium (Vector Laboratories, Burlingame, CA, USA) and analyzed using a Leica DMLB microscope equipped with a Leica DFC-480 camera (Leica Microsystems, Wetzlar, Germany). The control sections incubated while omitting the DIG-oligonucleotide probes and with sense oligoprobes displayed no color staining.

### Double-staining by ISH and GFAP immunostaining

To prove IL-6 mRNA signals in SGC, we used simultaneous fluorescence ISH and immunostaining of glial fibrillary acidic protein (GFAP). Digoxigenin from ISH was detected by incubation with an anti-DIG monoclonal antibody that was fluorescein isothiocyanate conjugated. The sections were then washed with PBS containing 0.05% Tween 20 (PBS-TW20) and 1% bovine serum albumin (BSA) for 10 min, treated with 5% normal donkey serum in PBS-TW20 for 30 min, and incubated with 25 μl of GFAP antibody (Dako, Glostrup, Denmark) in a humid chamber at room temperature (21–23°C) for 2 h. The GFAP immunoreaction was visualized by treatment with tetramethyl rhodamine isothiocyanate (TRITC)-conjugated and affinity-purified donkey anti-rabbit antibody for 90 min at room temperature. Sections of naïve DRG and those removed from rats operated upon to create CCI for both survival periods as well as from sham-operated rats were incubated simultaneously under the identical conditions. Immunostained sections were rinsed, stained with Hoechst 33342 to detect positions of the cell nuclei, then mounted in a Vectashield aqueous mounting medium (Vector Laboratories Inc, Burlingame, CA, USA). The control sections were incubated while omitting the primary antibody and displayed no immunostaining. The immunostained sections were observed and analyzed using a Leica DMLB epifluorescence microscope equipped with appropriate filter combinations and a Leica DFC-480 camera (Leica Microsystems, Wetzlar, Germany).

### Immunohistochemistry (IHC)

Sections of DRG from naïve and operated rats were immunostained simultaneously under the same conditions. They were washed with PBS containing 0.05% Tween 20 (PBS-T) and 1% BSA for 10 min, treated with 5% normal donkey serum in PBS-T for 30 min, and incubated with 25 μl of rabbit polyclonal antibodies against IL-6 (1:500; Invitrogen, Camarillo, CA, USA) or IL-6R (1:200; Santa Cruz Biotechnology, Santa Cruz, CA, USA) in a humid chamber at room temperature (21–23°C) for 12 h. The immunohistochemical reaction was visualized by treatment with TRITC-conjugated and affinity-purified donkey anti-rabbit secondary antibody (1:100; Millipor, Billerica, MA, USA) for 90 min at room temperature. The control sections were incubated without the primary antibody. Sections were mounted in aqueous mounting medium (Vectashield; Vector Laboratories Inc., Burlingame, CA, USA) and analyzed using an epifluorescence microscope (DMLB; Leica Microsystems GmbH, Wetzlar Germany) equipped with a camera (DFC-480; Leica Microsystems) and a stabilized power supply for the lamp housing.

### Statistical analyses

Behavioral data and data for staining density of IL-6 mRNA were evaluated using Kruskal–Wallis one-way analysis of variance and p values less than 0.05 were considered to be significant. All statistical analyses were performed using STATISTICA 9.0 software (StatSoft, Tulsa, OK, USA).

## Abbreviations

CCI: Chronic constriction injury; CNTF: Ciliary neurotrophic factor; DIG: Digoxigenin; DRG: Dorsal root ganglia; GFAP: Glial fibrillary acidic protein; LIF: Leukemia inhibitory factor; IL-6: Interleukin 6; ISH: In situ hybridization; SGC: Satellite glial cells.

## Competing interests

The authors declare that they have no competing interest.

## Authors’ contributions

VB created the experimental design, conducted ISH analyses, and wrote the manuscript. IK and ISH conducted surgical procedures. PD designed the studies and wrote and revised the manuscript. All authors read and approved the final manuscript.

## Supplementary Material

Additional file 1 **Control sections incubated with sense oligonucleotide probes displayed no color staining.** Detection by sense IL-6 probe (first lane, A-C), sense IL-6R probe (second lane, D-F), and sense GP130 probe (third lane, G operated (second and third rows, B, C, E, F, H, I) rats. Scale bars = 50 μm. H). Naïve DRG (first column, A, D, G) and DRG from CCI. Click here for file

Additional file 2 **Sections illustrating immunofluorescence staining for IL-6 protein in lumbar DRG from naive rat (A) and lumbar DRG from ipsilateral (B, L-DRGi) and contralateral side (C, L-DRGc) as well as cervical DRG (D, C-DRGn) of rat 3 d from CCI of the sciatic nerve.** Increased immunofluorescence was observed mainly in neuronal bodies of both lumbar and cervical DRG. Scale bars = 50 μm. Click here for file

Additional file 3 **Sections illustrating immunofluorescence staining for IL-6R protein in lumbar DRG from naive rat (A) and lumbar DRG from ipsilateral (B, L-DRGi) and contralateral side (C, L-DRGc) as well as cervical DRG (D, C-DRGn) of rat 3 d from CCI of the sciatic nerve.** Increased immunofluorescence was observed mainly in neuronal bodies of both lumbar and cervical DRG. Scale bars = 50 μm. Click here for file
