# Supplementary material for: Dynamic response to peripheral nerve injury detected by in situ hybridization of IL-6 and its receptor mRNAs in the dorsal root ganglia is not strictly correlated with signs of neuropathic pain
Source: Mol Pain. 2013 Aug 16;9:42. doi: 10.1186/1744-8069-9-42 (PMC3844395; doi:10.1186/1744-8069-9-42)
Supplement: Additional file 1 — Control sections incubated with sense oligonucleotide probes displayed no color staining. Detection by sense IL-6 probe (first lane, A-C), sense IL-6R probe (second lane, D-F), and sense GP130 probe (third lane, G operated (second and third rows, B, C, E, F, H, I) rats. Scale bars = 50 μm. H). Naïve DRG (first column, A, D, G) and DRG from CCI. [file 1744-8069-9-42-S1.pdf]

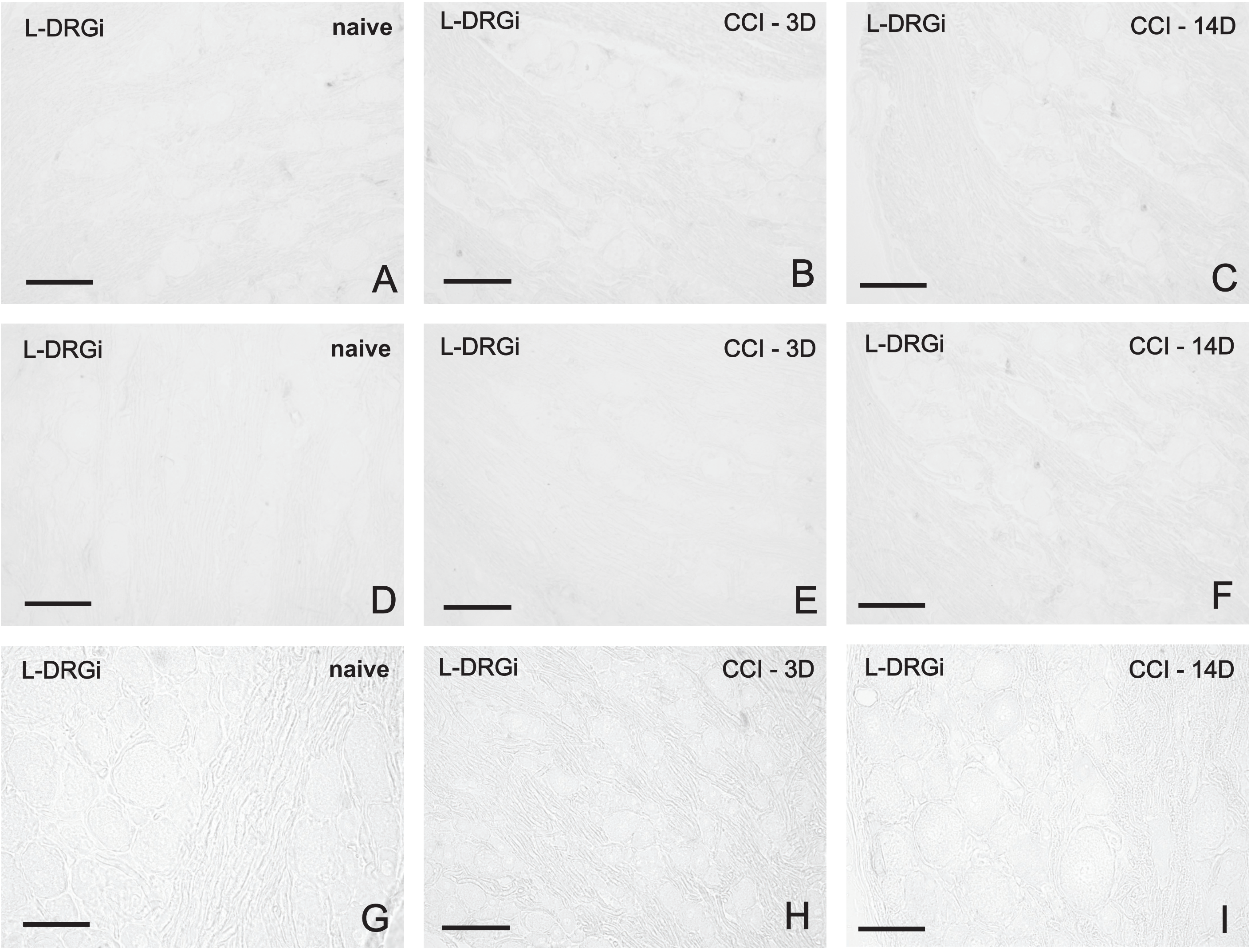

Additional file 1 Control sections incubated with sense oligonucleotide probes displayed no color staining. Detection by sense IL-6 probe (first lane, A-C), sense IL-6R probe (second lane, D-F), and sense GP130 probe (third lane, GH). Naïve DRG (first column, A, D, G) and DRG from CCI-operated (second and third rows, B, C, E, F, H, I) rats. Scale bars = 50 μm.
