# Supplementary material for: Dynamic response to peripheral nerve injury detected by in situ hybridization of IL-6 and its receptor mRNAs in the dorsal root ganglia is not strictly correlated with signs of neuropathic pain
Source: Mol Pain. 2013 Aug 16;9:42. doi: 10.1186/1744-8069-9-42 (PMC3844395; doi:10.1186/1744-8069-9-42)
Supplement: Additional file 2 — Sections illustrating immunofluorescence staining for IL-6 protein in lumbar DRG from naive rat (A) and lumbar DRG from ipsilateral (B, L-DRGi) and contralateral side (C, L-DRGc) as well as cervical DRG (D, C-DRGn) of rat 3 d from CCI of the sciatic nerve. Increased immunofluorescence was observed mainly in neuronal bodies of both lumbar and cervical DRG. Scale bars = 50 μm. [file 1744-8069-9-42-S2.pdf]

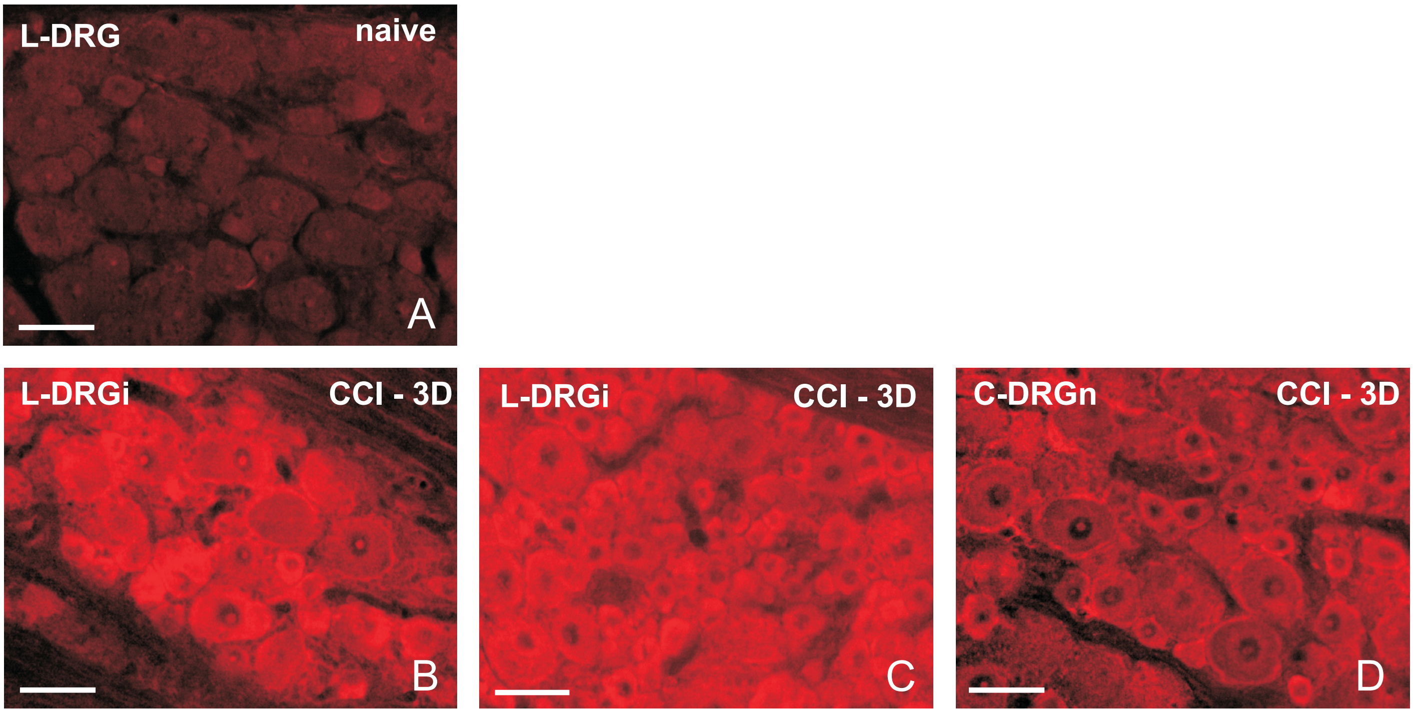

Additional file 2 Sections illustrating immunofluorescence staining for IL-6 protein in lumbar DRG from naive rat (A) and lumbar DRG from ipsilateral (B, L-DRGi) and contralateral side (C, L-DRGc) as well as cervical DRG (D, C-DRGn) of rat 3 d from CCI of the sciatic nerve. Increased immunofluorescence was observed mainly in neuronal bodies of both lumbar and cervical DRG. Scale bars = 50 µm.
